# Primary groin porocarcinoma in a 43‐year‐old man successfully managed by local excision: A case report and literature review

**DOI:** 10.1002/ccr3.7148

**Published:** 2023-09-14

**Authors:** Heba Megahed, Ayda Al‐Hammadi, Noura Al‐Nauaimi, Samir Al Hyassat, Mahmoud Al‐Thalathini

**Affiliations:** ^1^ Plastic and Reconstructive Surgery Department Hamad Medical Corporation Doha Qatar; ^2^ Dermatology Department Hamad Medical Corporation Doha Qatar; ^3^ Community Medicine Department Hamad Medical Corporation Doha Qatar; ^4^ Anatomical Pathology Department Hamad Medical Corporation Doha Qatar

**Keywords:** eccrine porocarcinoma, skin cancer, sweat gland tumors

## Abstract

Porocarcinoma is a rare skin malignancy that should be suspected in patients with chronic skin lesions. Although it usually affects the elderly population in sun‐exposed areas, it may be seen in younger populations and in nonexposed areas such as the groin.

## Graphical abstract

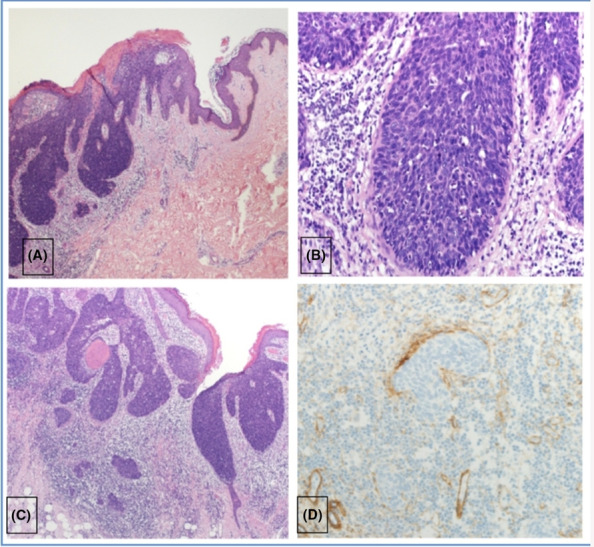

## 1 INTRODUCTION

Porocarcinoma is a rare skin cancer that usually affects elderly patients in the sixth and seventh decades of life with a predilection to affect the head, neck, and extremities. We report a rare case of porocarcinoma of the groin in a 43‐year‐old gentleman that was successfully managed by wide local excision.

Porocarcinoma is a rare skin cancer arising from the intraepidermal portion of the eccrine sweat glands.^1^, ^2^, ^3^ Although the cause of porocarcinoma is not well‐known, suggested risk factors for its development include a preexisting eccrine poroma, chronic phototoxicity, and chemical and radiation exposure in addition to immune suppression.^2^, ^3^ Porocarcinoma represents 0.005% to 0.01% of all cutaneous tumors, and the incidence of porocarcinoma in Europe was estimated to be <0.28/100,000^4^; however, it is considered the most common type of eccrine sweat gland cancers.^2^ To date, there are no data about the incidence of porocarcinoma in the Middle East and North Africa (MENA) region.

The largest review of porocarcinoma‐reported cases, which included 453 patients, showed that porocarcinoma occurs with equal gender predilection and a tendency to affect the elderly population more commonly in the seventh and eighth decades of life.^3^ Porocarcinoma commonly presents on the head, neck, or lower extremities; yet, it rarely affects the trunk.^2^, ^3^, ^5^ In a review of 206 cases, inguinal porocarcinoma was seen only in two patients.^2^ Porocarcinoma can appear in multiple forms. However, the most common presentation is a painless mass or nodule on the skin on top of a long‐standing lesion (poroma).^3^ The definitive diagnosis of porocarcinoma depends on the histopathologic examination of the tissue biopsy.^6^ Due to its rarity, there is no consensus on managing porocarcinoma. Nevertheless, wide local excision is the most common treatment for localized disease.^2^, ^3^ On the contrary, the metastatic disease showed more variable options, including wide local excision with lymphadenectomy, radiotherapy, or chemotherapy.^2^

We present a rare groin porocarcinoma in a 43‐year‐old gentleman successfully managed by early local resection. This case is peculiar because primary porocarcinoma is rarely seen in the fourth decade of life. In addition, only a few cases of primary porocarcinoma have been reported to affect the groin area.

## 2 CASE PRESENTATION

A 43‐year‐old Indian gentleman, previously healthy, presented to dermatology clinic complaining of a suspicious skin lesion on his right groin. The lesion started as a painless nodule 4 years ago and slowly increased in size. Four months before the presentation, the patient started to feel pain at the site of the lesion with pus discharge. There was no history of other swellings, previous illness, radiation exposure, loss of weight, or family history of cancer.

**FIGURE 1:**
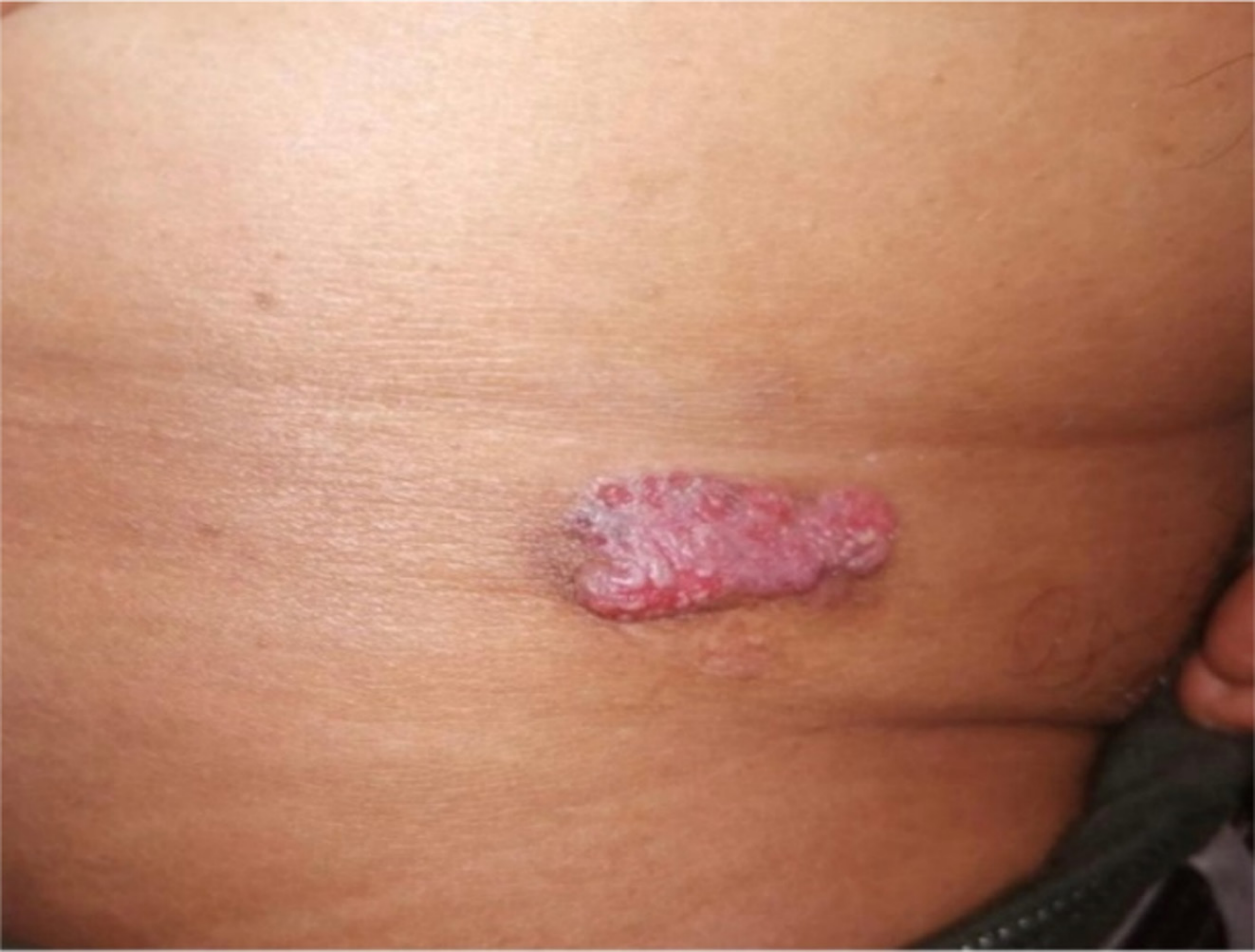
Groin porocarcinoma appearing as an erythematous 2 × 3 cm oval nodule on the right groin skin with superficial erosion.

On examination, there was a 2 × 3 cm oval erythematous ugly nodule on the right groin skin with superficial erosion and overlying pus discharge (Figure 1). The lesion was firm, fixed to the skin, and tender to palpation. Examination showed no lymphadenopathy or other skin lesions. The patient was counseled about the condition and the need for histopathological evaluation. An incisional biopsy of the nodule was performed under local anesthesia.

Histological examination showed marked full‐thickness atypia of the epidermal tissue. Large pleomorphic cells with prominent nucleoli and high mitotic activity were identified forming endophytic lobules that appeared to be of sweat duct origin, suggesting porocarcinoma in situ.

The case was discussed in the skin cancer multidisciplinary meeting, and the patient had wide local excision of the skin tumor with a 2‐cm safety margin followed by primary closure by the dermo‐oncology plastic surgeon.

**FIGURE 2:**
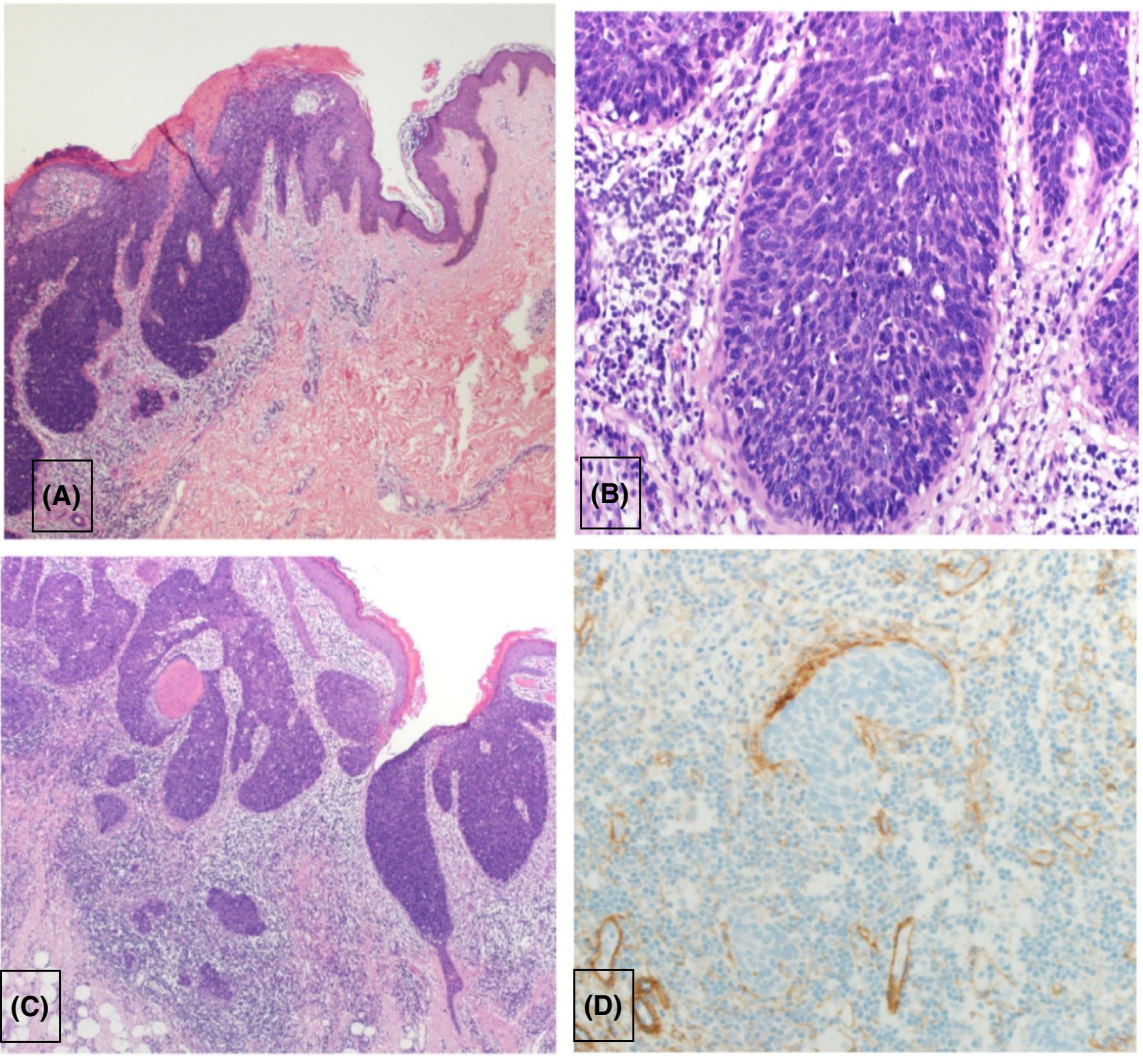
Histopathological examination of the lesion revealed full‐thickness dysplasia (A, H&E × 40) in keeping with a background porocarcinoma in situ. High‐power view highlights the presence of severely atypical basaloid cells with luminal formations (B, H&E × 200). Atypical tumor islands are noted in the mid‐dermis (C, H&E × 40), in keeping with invasive porocarcinoma. This has been confirmed by the demonstration of basement membrane breach using immunohistochemical staining for collagen 4 (D).

Histological examination showed marked full‐thickness atypia in the surface epithelium associated with luminal/glandular formations (Figure 2). Large pleomorphic cells with increased nuclear to cytoplasmic ratio, prominent nucleoli, and high mitotic activity were identified. Immunohistochemical staining for EMA highlighted the luminal formations. A diagnosis of porocarcinoma on a background of porocarcinoma in situ was made. The lesion was 2.9 × 1.4 cm clinically with invasion of the papillary dermis but no evidence of lymphovascular or perineural invasion. The carcinoma did not involve resection margins, and the disease was staged as pathological stage pT2 based on the Union for International Cancer Control (UICC) TNM 8 pathological staging for primary cutaneous carcinoma classification. The surgical site healing was uncomplicated. One‐year follow‐up showed no local recurrence. No chemotherapy or radiotherapy was given, and the patient is still under follow‐up.

## 3 DISCUSSION

The case we report is the first report in the MENA region of a groin porocarcinoma. This rare skin cancer usually affects the senior‐aged population's sun‐exposed areas and lower extremities.^3^ Porocarcinoma is rarely seen in the groin region and rarely affects young patients in the fourth decade of life, making this report of peculiar interest.

Eccrine porocarcinoma is a rare skin cancer originating from the intraepidermal part of the eccrine sweat glands. The tumor can either start de novo or on top of a previously benign lesion.^4^ In our case, the malignant lesion seems to complicate a long‐standing benign lesion that the patient has noticed for 4 years. Our review of the literature showed that the most common presentation is a solitary nodular mass on the head and neck region (32%), lower limbs (33%), trunk (14%), and genitalia (11.2%).^2^ Although porocarcinoma is usually asymptomatic,^3^ our patient sought medical advice after experiencing pain and discharge from his long‐standing silent skin lesion, which may suggest that a local infection was the inciting event for diagnosis.

Regarding the diagnosis, porocarcinoma has a myriad of differential diagnoses. The list includes metastatic adenocarcinoma, Bowen's disease, pyogenic granuloma, fibroma, seborrheic keratosis, and amelanotic melanoma.^1^ The definitive diagnosis is always based on the histopathological examination.

The histological appearance of porocarcinoma varies widely.^2^ However, nuclear atypia, with prominent hyperchromatic nucleoli and extensive mitosis, is common findings.^2^, ^7^ Intraepidermal cell nests of polygonal tumor cells can be seen. Cells follow an asymmetrical invasive, infiltrative growth pattern extending from the epidermis toward the deep dermis.^2^ No single immunohistochemical marker can identify eccrine cells. However, epithelial membrane antigen (EMA), carcinoembryonic antigen (CEA), cytokeratin, P 53, P 63 are used as part of the diagnostic panel to identify eccrine origin of cells.^2^, ^7^ In our patient, the histological changes were consistent with the above description, and immunohistochemistry was positive for EMA and CEA.

Wollina et al. evaluated the histopathological and immunohistochemical differences between poroma and porocarcinoma. In this study, porocarcinoma showed a widely variable response to cellular markers compared with benign poroma. Porocarcinoma showed strong CEA expression by lumen‐forming single vacuolated cells and mature duct cells. Such expression was limited in single poromas lumen formations. Porocarcinoma cell clusters showed scattered reactivity with antibodies Cam 5.2 and Vim 9(1), for which poromas remained unstained. In both poroma and porocarcinoma, scattered cells stained positive for S100A. These cells showed well‐formed dendrites in poroma, while porocarcinoma failed to show dendrites and showed irregularly shaped cells..^8^

As per the management, wide surgical excision is the most common treatment option for the local disease, with curative rates from 70% to 80%.^4^ In our case, a 2‐cm safety peripheral margin was removed, including a subcutaneous fat layer. Follow‐up for 1 year showed no local recurrence. No metastatic evaluation was made as the margins were not involved, and no lymphadenopathy was noted clinically. Our case stresses the importance of early diagnosis and management of such skin lesions, a benefit that can save the patient from further aggressive management.

## 4 CONCLUSION

Eccrine porocarcinoma is a rare skin malignancy. Although it is primarily seen in the head, neck, and extremities of elderly patients, it can be found in the younger population and nonexposed areas, such as the groin. Diagnosing this rare cancer is challenging, so clinicians should have a high index of suspicion when facing such lesions. It is crucial to reach an early histological diagnosis for this malignancy, as wide local excision can be the only intervention needed for the localized disease.

## AUTHOR CONTRIBUTIONS

**Heba Megahed:** Conceptualization; data curation; project administration; validation; writing – original draft; writing – review and editing. **Ayda Al‐Hammadi:** Data curation; writing – original draft. **Noura Al‐Nauaimi:** Data curation. **Sameer Alhyassat:** Data curation; writing – review and editing. **Mahmoud Al‐Thalthini:** Supervision; writing – review and editing.

## CONFLICT OF INTEREST STATEMENT

The authors declare no conflicts of interest with the subject of this article.

## ETHICS STATEMENT

Approved by Surgical Research Center, Hamad Medical Corporation REF: SR/RE/2022/41.

## CONSENT

Written consent for the publication of the clinical details of this case report is present upon request.

## Data Availability

Data available on request from the authors.
